# Online Knowledge Translation Program Involving Video Games and University Student–Led Tutorials About Cannabis and Psychosis for Black Youth: Mixed Method Feasibility Study

**DOI:** 10.2196/33693

**Published:** 2022-06-20

**Authors:** Payal Jani, Nuri Song, Erin Artna, Joonsoo Lyeo, Ashley Assam, Faith Maelzer, Andriene Murphy, Angelo Grant, Suzanne Archie

**Affiliations:** 1 Faculty of Health Sciences McMaster University Hamilton, ON Canada; 2 Department of Psychology, Neurosciences and Behaviour McMaster University Hamilton, ON Canada

**Keywords:** knowledge translation, Black youth, video game, psychosis, cannabis use, knowledge, young adult, race, demographic, minority, gaming, mental health, drug, cannabis, acceptability, feasibility, risk

## Abstract

**Background:**

We have piloted a new online knowledge translation (KT) program created to educate youth about cannabis effects, which uniquely focuses on mental health risks for Black youth. Youth are generally unaware of the research linking underage usage and the risk of psychosis. Youth from some Black racialized communities in Ontario may be disproportionately affected and in need of this knowledge.

**Objective:**

Because very little is known about the acceptability and feasibility of programs educating Black youth about cannabis and psychosis risk, we evaluated this KT program, which consists of tutorials facilitated by university students and video games.

**Methods:**

This mixed methods pilot study evaluates the transfer of knowledge about cannabis and psychosis risk before and after the online KT program and, at the same time, explores participant satisfaction with the program and views about underage use. Eligible participants were youth 16-19 years of age of Black African or Caribbean descent. Trained undergraduate students from McMaster University administered a quiz (psychosis and cannabis test; PCT) to evaluate knowledge before and after the KT program. After playing the psychoeducational video games, participants attended two tutorial group sessions led by undergraduate students. The undergraduate students facilitated the online tutorials about cannabis and psychosis. The tutorials augmented the educational content embedded within the gameplay: participants discussed what they learned from the video games and their understanding of psychosis and the effects of cannabis. In addition, undergraduate students qualitatively analyzed the tutorial discussions for themes, and the prequiz and postquiz scores were analyzed for significant differences in scores.

**Results:**

A total of 9 Black youth were recruited and completed this pilot study. The mean PCT scores were 5.67 (SD 1.7) and 7.78 (SD 1.8) before and after the KT program, respectively. There was a significant improvement in scores (*P*<.05) post-KT program. Thematic analysis of the facilitated tutorials revealed three major themes: video game satisfaction, marijuana and psychosis literacy, and help-seeking awareness. Overall, participants showed an increased awareness and understanding of the subject matter after the gameplay and tutorial intervention.

**Conclusions:**

When supplemented with tutorial sessions, the Back to Reality Series shows promise for addressing the gap in knowledge about cannabis and psychosis, and the results provide preliminary evidence that the games appeal to Black youth.

## Introduction

### Background

Before its legalization for nonmedical use in 2018, cannabis was the most widely used illicit substance in Canada. Among advanced economies, Canada has a relatively high rate of adolescent cannabis use [1], as high as 28% among youths between 15 and 19 years old [2]. Given policies to restrict cannabis use among underage youth, one might expect greater public education geared toward youth. In a 2017 Canada-wide survey, less than 50% of youth could identify the mental health effects of cannabis [3,4]. Gaps in knowledge about risks of underage use have also been noted in the United States [4].

Even though the majority of healthy adult cannabis users do not experience health risks from use [5], studies have found a significant relationship between underage cannabis consumption and the onset of subsequent psychosis [6-8]. Several risk factors influence this relationship: the age of onset of regular use [8], frequency of use [8], genetic vulnerability for schizophrenia [9,10], previous psychosis symptoms [11], genetic risk for schizophrenia, and the delta-9-tetrahydrocannabinol (THC) content or potency of the cannabis consumed [11]. THC is the psychoactive component of cannabis associated with addiction and hallucinatory experiences [12]. The higher the THC content, the greater the risk of experiencing these effects [8]. Underage cannabis use may pose additional barriers for youth from Black racialized communities.

There are few public health tools about cannabis use and its mental health effects (eg, Canada’s Lower-Risk Cannabis Use Guidelines [13]), but generally, public education about the mental health effects of cannabis for youth is lacking, particularly in Black communities. Black youth have experienced disproportionate rates of criminalization, stigma, and negative stereotypes associated with cannabis use [14]. Credible knowledge delivered in a culturally safe manner is needed for this population. A recent Ontario study suggests that people from Black Caribbean communities versus Canadian-born respondents had higher rates of past-year cannabis use (odds ratio [OR] 1.70, 95% CI 1.04-2.79; *P*<.01). Furthermore, this group’s risk was also higher (OR 2.76, CI 1.24-6.12; *P<.*05) for problematic cannabis use—defined as a pattern of use associated with harm, abuse, or dependence [15]. In contrast, for other Black ethnic groups, the OR was lower for people from the Black African group for past-year use and was not significantly different from Canadian-born respondents (OR 0.68, 95% CI 0.35-1.31) [15]. Black racialized communities do not represent a monolith. Interventions designed explicitly for Black youth are rare [16], and even these could benefit from innovative strategies that appeal to diverse ethnic groups within Black communities.

### Interactive Tutorials and Peer Modelling

Interactive tutorials are a promising educational method compared to traditional lecture-based or textbook instructions [17]. Active learning techniques are associated with higher skill acquisition and improved motivation for learning (eg, use of interactive video games, informal games, and in-class time devoted to discussions about a case) [17]. University student–led tutorials for more junior learners offer peer role modeling and may also enhance professional and communication skills [18]. The undergraduates learn to explain the scientific knowledge, and the participants are encouraged to engage in critical thinking about the science [18]. Combining multimedia strategies such as animation with community-based programming offered by schools or family physician’s offices has augmented behavioral change, even reducing illicit drug use [19].

### Serious Video Games

Online digital video game technology offers engrossing platforms for youth to receive and integrate mental health information [20]. Immersive and interactive story lines have been shown to promote health-related behavioral change and facilitate deep learning [21,22]. Therapeutic video games are an emerging area in the health care field because of their capacity to simulate real-life symptoms and treatment [23]. Video game technology has improved outcomes for US Army veterans with posttraumatic stress disorder [24]. Sparx, a video game that uses avatars to deliver cognitive behavioral therapy (CBT), is effective for depression using a randomized control design [25,26]. Reductions in paranoid ideations and anxiety have been achieved for patients with psychosis compared to treatment as usual, using immersive virtual reality CBT for patients [27]. Nonetheless, media campaigns that warn youth about the harms of substance use, particularly tobacco or illicit drugs, have yielded mixed results [28].

### The Back to Reality Video Game Series

The Back to Reality Video Game Series (the SERIES), a knowledge translation (KT) product, was created to translate messages inspired by research on cannabis use [29,30] and pathways to care for a first episode of psychosis for young people of Black African and Caribbean descent [31,32]. The games were produced with input from an integrated KT community of Black youth, students, young people with lived experiences, family members, game designers, and researchers. It was created using principles of serious game design methodology for effective game design in education [33]. Interactivity helps players explore the potential benefits, harms, and emotional, social, or psychiatric consequences of regular cannabis use. It depicts Harry and his group of friends from diverse ethnic backgrounds exploring the potential positive and negative consequences of cannabis use. Harry is an 18-year-old second-generation Canadian youth of Jamaican descent who develops psychosis after regular cannabis use and enters a virtual mental health system as a result. It “shows” rather than “tells,” using a medium appropriate for the audience. Family physicians thought the SERIES met their needs to educate young people about cannabis use, mental health, and addiction services [34]. They expressed a willingness to offer it to young people with mental health and addiction issues and their families [34].

These studies support the use of serious video games and interactive learning strategies to address mental health and addictions issues. Nevertheless, the existing literature lacks data on interventions designed to help youth from Black communities understand the mental health impacts of cannabis. Black youth from Caribbean communities may be more at risk for problematic cannabis use [15] and more vulnerable to the impact of stigmatization and criminalization [14]. However, very little is known about how to translate research knowledge for this population. In this pilot study, Black youth were exposed to video games and university student–led tutorials to evaluate the feasibility and acceptability of this KT program about cannabis and psychosis. The participants are expected to acquire relevant knowledge about underage cannabis use and the risk of psychosis. This paper explores themes emerging from the tutorial sessions about participant satisfaction with the KT program. This evidence could spur future randomized control trials on the effectiveness of educational and online digital strategies for increasing learning outcomes about the mental health impacts of cannabis.

## Methods

### Overview

This project collected data on a new KT program involving psychoeducational video games and tutorials designed to educate Black African and Caribbean youth about the relationship between cannabis and psychosis. Our objective was to explore the extent to which this online KT program could transfer health information to youth—a challenging population to engage in medical literacy—and assess user satisfaction. This pilot project was based on a partnership involving McMaster University and the Free for All Foundation, a community charitable organization in Ontario that serves Black youth and their families. It is a grassroots community-based program that provides support so that Black youth can fulfill their potential.

The study used qualitative and quantitative data collected before, during, and after two tutorial sessions facilitated by undergraduate students. The primary variable was changes in scores on a knowledge test, the psychosis and cannabis test (PCT) quiz. The training was conducted from September to December 2020, and the recruitment, data collection, and analysis occurred between January and April 2021.

A group of 10 undergraduate students at McMaster University participated in a research-based thesis course under the supervision of the senior author. Over 4 months, these undergraduate students studied the research relevant to the association between youth cannabis use and psychosis, using the Back to Reality Series as the inspiration for their learning objectives. They were taught how to facilitate tutorial sessions. The undergraduate students were trained in the proper conduct of data collection, transcribing the data, analyzing, coding procedures, generating themes, and writing up the report. This manuscript is based on input from many of the undergraduate students supervised by the senior author.

Convenience sampling was used to recruit Black youth from the community organization. The inclusion criteria were male, female, or transgender Black youth registered at the community organization and aged between 16 and 19 years. Youth were excluded from the study if they had a known seizure disorder or a video game addiction. Before their inclusion, each participant was administered a screening tool to assess their eligibility.

### Ethical Considerations

This study was approved by the Hamilton Integrated Review Ethics Board (11181), and each participant provided written informed consent for participation.

### Interventions

The online KT program consisted of two parts: tutorial sessions facilitated by undergraduate students and the Back to Reality Series video games.

### Tutorial Sessions

There were 3 tutorial groups. Each group underwent two online tutorial sessions, each led by 3-4 undergraduate students. Tutorials lasted 60 minutes each and were conducted over Zoom. The undergraduate students translated the scientific knowledge about cannabis and psychosis they had acquired the semester before into simplified teaching content for the participants. The learning objectives were to increase the participants’ awareness of the benefits and harms of cannabis. The goal was to help participants understand why underage cannabis use carried a greater risk of adverse consequences than adult-onset use, based on scientific research. Other learning objectives were as follows:

- Understand psychosis disorders and cannabis use disorder
- Appreciate factors mediating the risks associated with underage cannabis use and psychosis, including the following:
  Age of first use
  Potency of marijuana
  Genetic vulnerability
  Choice of products
  Pathways to care for marijuana use disorder and psychosis

The learning objectives were inspired by the narratives explored within the video game play, and the undergraduate students conferred with each other to ensure similar content was covered across tutorial groups. All participants played the SERIES, establishing a common ground for educational content. Active learning strategies such as interactive games, discussions, and problem-based learning incorporating narratives from the gameplay were incorporated into the tutorial sessions facilitated by the undergraduate students. Participants were able to discuss their experience playing video games and have their questions answered.

### Back to Reality Series

**Figure 1:**
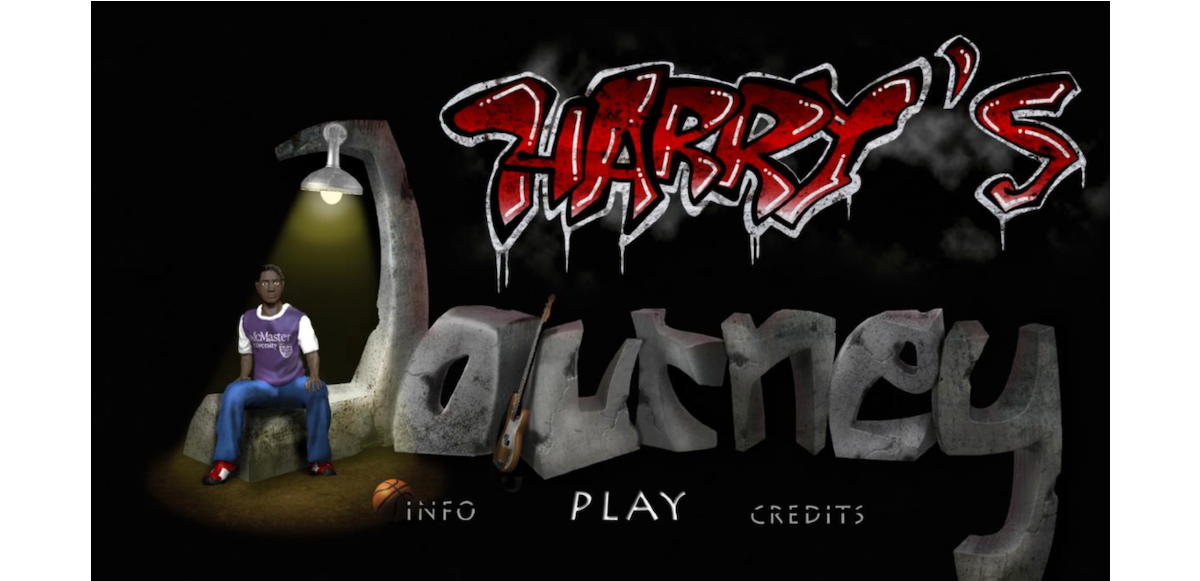
Harry's Journey main menu scene.

The SERIES consists of 3 video games: Harry’s Journey, Harry’s Journal, and Harry’s PathwaysToCare map. Harry’s Journey imparts the story of Harry—as he considers whether or not to seek help for his psychosis and cannabis use (Figure 1). Harry’s Journal delivers experiential knowledge about major psychiatric symptoms. The PathwaysToCare Map displays 3D replicas of youth mental health and addictions services so that young people can learn about the health care system and how to access care. McMaster University owns the intellectual property.

In a demonstration project [35], 20 undergraduate university students played the SERIES and a control game. All participants played both games, but the order was randomized within the software to determine which game was played first. The participants completed a quiz after playing each game. Playing the control game first, followed by the SERIES, led to significant increases in the quiz scores (*P*=.005). Participants randomized to playing the SERIES first, followed by the control game, had no significant changes in quiz scores, suggesting the SERIES was effective in transferring knowledge, but the control game was not.

The SERIES has been pilot tested among youth aged between 16 and 19 years and experiencing homelessness: 45% (25/55) were neither in school or working, 24% (13/55) reported psychosis experiences, and 13% (7/55) were of Black African/Caribbean descent. Furthermore, 88% (48/55) of the sample had a lifetime use of cannabis, with an average cannabis use onset of 13 years. This pilot study involving 55 youth demonstrated a significant mean knowledge test score advantage for participants playing the SERIES (54%; *P*=.02; mean 6.7, SD 1.7) compared to those playing the control game (mean 5.5, SD 2.0) [36]. The SERIES yielded a 22% improvement in test scores compared to the control game. The majority of participants (49/55, 90%) playing the SERIES enjoyed it versus 75% (41/55) of those playing the control game. Furthermore, the qualitative analysis revealed that Harry’s mental health experiences resonated with them, with participants saying “I felt like I knew what he was going through” and “Um, a lot of people make it run their life, and they skip school…like weed of all things their top priority.”

During preliminary testing, 10 participants aged between 17 and 30 years with a first episode of psychosis felt that Harry’s Journey realistically portrayed psychosis experiences, and they enjoyed playing it [37].

### Measures

Demographic data were collected on age, gender, and highest academic achievement.

#### PCT Quiz

The PCT quiz is a test of knowledge for participants. It does not involve clinical judgment or measurement of a clinical condition. The PCT quiz was constructed and validated to examine whether the Back to Reality Series delivers knowledge about the relationship between psychosis and cannabis (Multimedia Appendix 1). It consists of 10 multiple-choice knowledge questions worth 1 point each. The questions were reviewed, pilot tested, and revised by a 15-year-old high school student and a psychiatry resident who were part of a KT community that constructed and evaluated the video games. Items were generated from concepts arising out of the literature review concerning the relationship. When administering the quiz, student researchers read the questions aloud to reduce the impact of reading literacy levels. It takes 10 minutes to complete the quiz.

The PCT quiz has produced consistent results supporting its reliability—replicating significant differences in knowledge acquisition scores after exposure to the SERIES across 3 small-scale demonstration projects. In 2020, a group of 20 McMaster University undergraduate students aged 17-22 years played two games, Morpheus Spell (a control game) and the SERIES [35]. The order in which the students played the game was randomized, but they all played both video games and completed the same two quizzes after playing each game. The participants were administered quiz 1 (a more challenging version of the PCT quiz for older youth) and quiz 2 (the PCT quiz). Participants had significant increases in scores on both quiz 1 and the PCT quiz when they played Morpheus Spell first, followed by the SERIES. Both quiz 1 and the PCT quiz produced significant increases in scores when participants played Morpheus Spell first, followed by the SERIES: on quiz 1 the scores were 5.44 (SD 1.51) versus 6.78 (SD 1.48, *P*<.03), and for the PCT quiz the scores were 6.67 (SD1.41) versus 8.22 (SD 1.30, *P*<.005). Statistical significance was not achieved for either quiz when participants played the SERIES first, followed by the control game (quiz 1: *P*=.17 and PCT: *P*=.28). The control game did not transfer any knowledge relevant to cannabis use or psychosis. This latter finding validates the use of the PCT quiz to test knowledge acquisition.

The PCT quiz was pilot tested on 10 clients (aged 17-30 years) with a first episode of psychosis using a pre-post design [37]. The pre-PCT quiz mean score was 6.5 (SD 1.3) versus the post-PCT quiz mean score of 7.7 (SD 1.4), revealing a significant increase (18% improvement) in posttest scores (*P*<.01), supporting its face validity [37]. The PCT quiz was also used with 55 youth experiencing homelessness who were randomized to either the SERIES or the control game first, as described above [38], revealing a statistically significant improvement (>18% difference) in scores (*P*<.05).

#### Postgameplay Survey

Participants were given a postplay survey consisting of 10 questions that gathered information on how often they play video games, which devices they use, their level of comfort while playing the Back to Reality Series, whether they enjoyed different aspects of the game (recommend game to a friend; thought game was youth-friendly; and enjoyed the story, the music, the graphics, the basketball minigame, and the game as a whole). The satisfaction questions were scored yes (1) or no (0). The survey was adapted from a study examining the acceptability and satisfaction with a video game promoting messages and actions concerning antenatal care [39].

### Procedures

Participants took part in 3 online zoom sessions conducted by undergraduate students. During visit 1, the participants answered structured demographic questions and open-ended questions about their understanding of cannabis and psychosis. Undergraduate students administered the PCT quiz to establish the participants’ knowledge base. Next, undergraduate students shared their screens with participants who played Harry’s Journey over Zoom. The subsequent two sessions involved tutorials led by the university students. All tutorial discussions were recorded over Zoom. After the first tutorial (visit 2), participants played the remaining games—Harry’s Journey and the PathwaysToCare map. After the second tutorial (visit 3), participants completed the postplay survey to assess their satisfaction with gameplay. Participants answered qualitative questions about their gameplay experience and their views on cannabis. The PCT quiz was readministered to assess changes in their level of knowledge.

All responses were recorded and transcribed by the undergraduate students. Identifiers were removed from each transcript and amalgamated into one encrypted document for thematic analysis by undergraduate students and the senior author.

### Data Analysis

#### Statistical Analysis

Chi-square and *t* tests were used to analyze the demographic and pre-post gameplay survey. A mean and standard deviation were calculated for participants’ PCT scores before and after the gameplay and tutorial intervention. A 2-tailed paired *t* test was then conducted using the before and after PCT scores to determine whether there was a significant change in scores. A *P* value <.05 was deemed significant for this statistical test.

#### Qualitative Analysis

The tutorial sessions for all groups were transcribed and consolidated into one document for all the undergraduate students to analyze. Each undergraduate student independently analyzed the data corpus, using the thematic analytic method espoused by Braun and Clarke [40] to identify patterns and meanings from data extracts and initial codes. Each student independently coded the data into themes and a thematic map. Two authors (PJ and SA) reviewed these codes and data extracts and selected the most poignant. The final descriptive thematic map organizes and highlights the prominent themes and subthemes.

## Results

### Overview

There were a total of 9 participants. Table 1 outlines the demographic characteristics of the 9 Black participants. The majority of participants (7/9, 77%) were male and attending or recently completed high school. There was complete attendance for all 3 visits.

**Table 1:** Participant demographic data (N=9).

Variable			Values
Race, Black, n (%)			9 (100)
Age (years), mean (SD)			17.56 (1.17)
**Gender, n (%)**			
	Male	7 (78)	
	Female	2 (22)	
**Educational enrollment, n (%)**			
	High school	5 (56)	
	Postsecondary	2 (22)	
	Not in school^a^	2 (22)	

^a^Completed high school.

### PCT Quiz

Table 2 shows a significant difference in PCT quiz scores before (mean 5.67, SD 1.66) versus the post-PCT quiz score (mean 7.78, SD 1.79) after the KT program (*P*=.01). Overall, 7 participants (77%) showed improvement in their PCT scores, and 2 (22%) participants had scores that remained unchanged.

**Table 2:** Knowledge translation program scores before and after participation.

Statistic		Values
**Score, mean (SD)**		
	Before	5.67 (1.66)
	After	7.78 (1.79)
**95% CI**		
	Before	4.58-6.75
	After	6.61-8.95
*P* value		.01

### Postgameplay Survey

**Figure 2:**
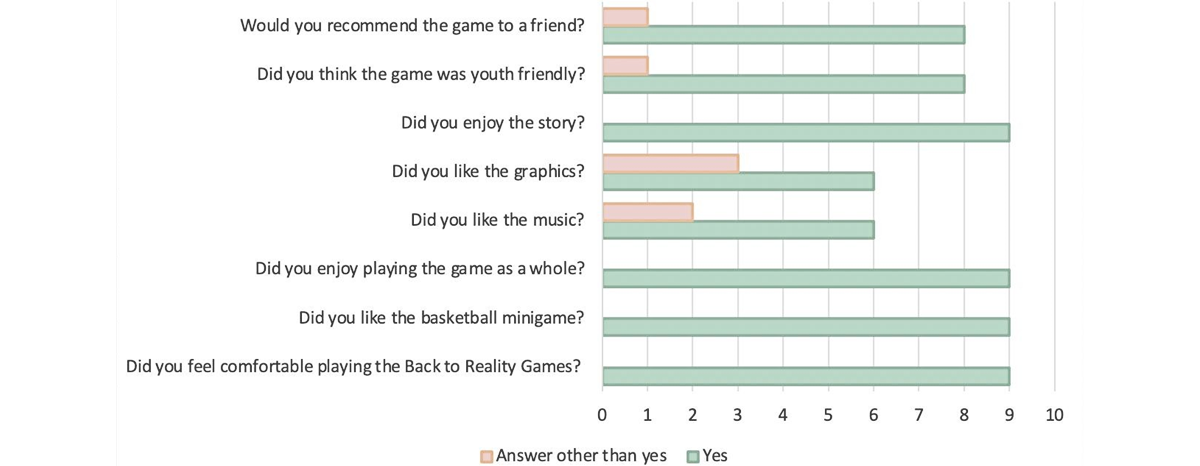
Postplay survey of gameplay satisfaction.

The majority of participants (5/9, 56%) played video games at a frequency of less than once per month. Smartphones were the most popular device used for gaming. Figure 2 outlines satisfaction with the Back to Reality Series based on the postgameplay survey. Participants unanimously reported they enjoyed the story, gameplay experience, and basketball minigame. The least satisfactory elements were the graphics and the music. The criticisms were reported by participants who played video games “almost every day” or “at least once a week.”

### Qualitative Analysis

Tutorials supported further deliberations about video games in an environment where participants felt supported by postsecondary students. Participants were given a chance to hear from other youth and formulate answers to questions. Participants were often seen learning from one another, adding to each other’s answers, and engaging in friendly competition during interactive activities.

**Figure 3:**
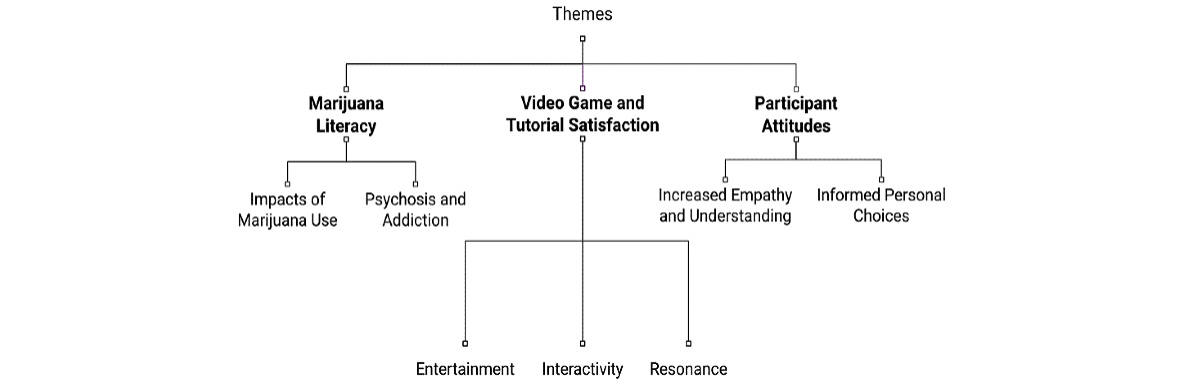
Thematic analysis map of tutorial discussions and video game feedback.

Discussions from the transcribed tutorials and qualitative interviews were classified into three major themes: (1) video game and tutorial satisfaction, (2) cannabis and psychosis literacy, and (3) participant attitudes. Under each central theme, 2-3 subthemes were identified, shown in Figure 3.

### Video Game and Tutorial Satisfaction

The first theme encompassed participants’ opinions about playing the SERIES and attending the educational tutorials. Several elements of the intervention were discussed; however, participants’ comments primarily reflected the degree of entertainment and interactivity they experienced when playing the video game. Furthermore, participants identified with the main character and experienced the narrative as authentic.

#### Entertainment

When asked to characterize their experiences with the game, many participants commented on the dualities of entertainment and education, suggesting entertainment increased engagement in their learning. Additionally, participants frequently used terms such as “fun,” “enjoying,” “entertaining,” and “like” to describe their experience with the intervention, showcasing a positive attitude toward it (eg, “It was actually fun. I enjoyed the game, and I also learned a lot too… It was a great experience”).

In the postgameplay survey, 100% (9/9) of participants said that they enjoyed playing the Back to Reality Series as a whole. The surprise element of the mental health experiences helped, particularly when the plot contrasted with their expectations and knowledge.

Yeah, I would because, uh it’s a visual way to represent what someone in that situation would be going through, and I feel like maybe someone who does use marijuana, then it can be a sort of visual way for them to see someone who its affecting negatively. Yeah, this would be awesome.

#### Interactivity

The KT program integrated interactivity heavily through gameplay involving the video games and open-ended discussions during tutorials. This interactivity was consistently noted by participants, who suggested its importance in their understanding of concepts (eg, “I liked the interacting [tutorial] games that we did, it was fun. Because we were playing games but also learning”).

Additionally, the first-person point of view of the video game may have led to increased personal investment in the outcomes of decisions. Since these outcomes revealed information about cannabis-associated risks, this engagement may have facilitated learning.

In terms of the game, I really like the fact I was able to control the decisions that Harry made cause then I can see how based on those decisions how it affected his life. And yeah...my biggest takeaway was really the decision-making aspect.

#### Resonance

The story line in the SERIES included many elements of teenage life, such as school, relationships, and families. As a result, many participants reported feeling more connected to the intervention, increasing engagement. Moreover, some participants discussed having friends who used cannabis and reported that the content was meaningful to their lives (eg, “I guess the fact that like Harry lived a realistic teenage lifestyle that like a lot of youth can relate to and that made it a lot more meaningful cause it felt more relevant”).

When asked if they would recommend the game to a friend, one participant stated, “Yeah…Because they actually learn and see kind of like, um, like a reflection of themselves.”

### Cannabis and Psychosis Literacy

The transcribed data set revealed a significant improvement in cannabis and psychosis-related knowledge among participants postintervention. All 9 participants described their participation in the intervention as educational or informative. Participants primarily acquired knowledge about the impacts of cannabis use, psychosis, and addiction.

#### Impacts of Cannabis Use

Thematic analysis revealed that participants had a sound understanding of addiction before starting the KT program (one participant said “Addiction, like it’s not a hobby, it’s more extensive than a hobby like it’s something you do commonly because you can’t stop”).

However, mental health risks were a prevalent but novel point of discussion in their postintervention interviews, suggesting newly emerged awareness achieved through their participation. Participants comment on the gravity of these mental health risks, adding nuance to this awareness: “Looking at it through like a mental lens, it could lead to like, dependency and addiction, um, which is also like a very big, um, mental health problem.”

Similarly, preintervention transcripts showed participants primarily discussing the social benefits of cannabis use and neglecting its social harms. Postintervention, there was a substantial consideration for these harms, with participants noting how in the Back to Reality Series, Harry’s relationships with his girlfriend, mother, and friends were negatively impacted due to his cannabis use and emerging psychosis (one participant noted “It might ruin friendships…[Harry] was paranoid with his social life he thought everybody was out to get him”). This direct reference to the video game resulting in their increased knowledge showcases its effectiveness as a multimethod educational tool.

#### Enhanced Psychosis and Addiction Knowledge

Prior to gameplay and tutorial intervention, 66.7% (6/9) of participants indicated that they did not know or were unsure of the meaning of psychosis.

I kind of learned a lot from it and the effects [marijuana] has. And also, like what psychosis is, because before when the game started, I didn’t know anything about psychosis.

Post-KT, participants were able to define psychosis and even distinguish between delusions and hallucinations, showing a nuanced understanding of the subject. There was a new awareness of the mental health risks of cannabis. Participants showed a strong understanding of the risk factors leading to cannabis addiction, psychosis, and the signs of addiction among cannabis users.

Someone that has psychosis will have lots of hallucinations—auditory, visual—and it will affect your perception of like reality.

I think it makes sense that like early-onset…would increase your risk of psychosis and addiction.

A sign that a youth is using marijuana to excess is that] they’re losing touch with their friends, isolating themselves, staying at home, not talking to anybody…being antisocial.

### Participant Attitudes

As a result of their newfound knowledge, a shift in attitude was seen among participants. Their postintervention comments reflected an increased sense of empathy and understanding for those suffering from cannabis addiction and seeking help. Additionally, participants became more self-reflective regarding their own cannabis use choices.

#### Increased Empathy and Understanding

Many participants expressed how their understanding of cannabis use became more balanced after playing the Back to Reality Series and attending the tutorial sessions. Some acknowledged how this challenged their previous misconceptions regarding cannabis users and allowed them to minimize their judgments toward this population.

Before coming into here I already had a bad connotation of marijuana because I just viewed it as bad…Then I played the game and came to these Zoom meetings with you guys, and I learned even more about why it's bad and what it can do for you, both the good and the bad.

Additionally, despite concerns about stigma, the importance of help-seeking for symptoms was a key message identified by some participants. Participants linked getting professional help to positive mental health consequences as opposed to negative consequences associated with delays. Many credited the video games with normalizing the process of seeking help, challenging pre-existing hesitancy they had about pathways to care for mental health and addiction issues.

Getting help is not something to be embarrassed about, it’s something that can actually help you…And the experience that [Harry] didn't...he got worse when he didn’t get help.

I guess when he got help at the support center because they didn't come off as it was his fault, they came off as...we address the situation, how can we help you now.

#### Informed Personal Choices

Four participants (44%) explicitly weighed out the pros and cons learned from the intervention to inform their decision-making (for their own and a hypothetical friend’s cannabis use). By linking knowledge to personal conclusions, the intervention is seen to impact their choices.

I personally think it’s…not necessary to be using [marijuana], especially considering the risks. I just think the cons outweigh the pros.

All the side effects...all those people who were originally affected went to those support groups…what they went through before they actually got the help they needed. And, like, based off that, that should tell you that [your friend] shouldn’t do

marijuana

## Discussion

### Principal Findings

Our results suggest that the KT program was acceptable and feasible for addressing the knowledge gap about cannabis and psychosis experienced by predominantly underage Black youth. This small-scale pre-post design suggests the KT program can be used with Black youth to increase their knowledge of cannabis and psychosis risk among youth. The tutorial discussions suggest that the participants were satisfied with the KT program, which they found entertaining, relevant, and educational. The video games resonated with their personal experiences. Our approach was novel because it employed peer modeling—undergraduate students, youth in their own right with a degree of academic success, facilitated complex discussions with Black youth about scientific data. Further work is needed, but the facilitated tutorials may allow participants to gain further insight beyond what was presented in the game. Youth-driven collaboration may be a motivating factor in encouraging discussion and engagement. The online format worked well and provided opportunities to engage youth with a 1:1 facilitator/participant ratio.

### Limitations

There were several limitations associated with this pilot project. Only 9 participants were enrolled in this study, which limits the generalizability of its results. The ethnic identity of the Black youth was not captured (Black Caribbean ethnicity versus Black African) in light of the small sample size. This flaw ignores significant differences in cannabis use among different ethnic groups in Black racialized communities [15], and these groups may respond differently to the same strategies.

Although the overall objectives for the tutorials were the same for each group, the facilitators had autonomy over the delivery of the tutorial content, so participants in different groups did not have the same tutorial content. However, the learning objectives were consistent among tutorial groups. The university students were trained as a group and conferred to ensure the tutorial content was similar between tutorial groups. The foundation of the knowledge—the video games—was consistent.

Most participants (8/9, 88%) reported using their smartphones to play video games, so creating a version for mobile phones might increase its accessibility. The reported frequency of video game play in the past year was low among participants. Approximately half of the participants (5/9, 56%) reported playing video games less than once per month in the past year. More specifically, the Ontario Student Drug Use and Health Survey found that among students in grades 7-12, 16.7% reported that they did not play video games, 22.6% played 3 times per month or less, 6.2% played once per week, 17.3% played 2-3 times per week, 13.0% played 4-5 times a week, and 24.3% played daily or almost daily [41]. Canadian youth who play video games more frequently might not be as impressed with the novelty of the SERIES and might expect graphic quality in keeping with commercial video games. The Back to Reality Series is a research prototype created with limited funding from research grants, hence the graphics and music are not on par with those typically found in commercial video games. Furthermore, our prototype could only be used with a Windows operating system. Additional minigames throughout gameplay might further increase user engagement and help with information recall. However, the entertainment value of the SERIES would be in competition with traditional psychoeducational programs about cannabis and mental health effects because it is geared for use by community agencies and mental health programs.

The design of this pilot fails to identify which component of the KT program had the most significant impact—the SERIES versus the tutorial sessions. Nevertheless, this KT program achieved a 37% increase in scores. This is the highest increase in scores among all of the demonstration projects and the only study to include tutorials. Therefore, the capacity of the SERIES to transfer knowledge appears to be augmented by the tutorial format. However, future research needs to establish whether the tutorial sessions enhance the learning provided by the games. The findings need to be replicated in a larger study to determine whether this KT program can be successfully implemented among a more extensive sample of Black and diverse youth populations.

The satisfaction questions may have introduced social desirability bias (“Did you like….?”). A Likert scale to assess satisfaction would introduce less bias than “Yes” and “No” responses to questions about video game satisfaction. Despite these shortcomings, it seems that participants enjoyed the gameplay experience overall based on their qualitative responses.

### Comparisons With Prior Work

Video games have been shown to simulate real experiences that may help players practice decision-making and actions needed for their real life by using gamified conditions to augment learning [42]. The participants valued the entertainment and educational components of the KT program. Video games have great appeal and are widely used by youth with serious mental illnesses [43]. Our video games seemed to ground the tutorial’s content with concrete examples and thus provided opportunities to consolidate concepts introduced by the games. Participants were able to engage in rich, student-led discussions by referring to narratives from within the video games without resorting to personal confidential information. The video games seemed to be engaging and succeeded in capturing the participants’ interest.

The context or setting for gathering information was very relevant for this study. Peer collaboration seemed to be a motivating factor in encouraging discussion and engagement. The online format worked well and provided opportunities to engage youth on a 1:1 basis with university student facilitators. A 2018 study involving undergraduate students revealed that interactive online tutorials with student-led discussions and self-assessment quizzes were more effective for knowledge transfer than traditional textbook learning [17].

### Conclusions

Effective strategies to educate youth about substance use risk have long been a challenge. This KT program adds a potential strategy to the list of online educational curricula. This study provides valuable insights into the feasibility and acceptability of this innovative KT program in disseminating cannabis-related health information to Black youth in Canada. Future research could increase the sample size and disaggregate the video game data from the tutorial sessions to understand the role played by each approach in educating Black youth. The KT program could also be studied in a larger, long-term trial to test whether this knowledge acquisition leads to less problematic cannabis use among youth.

## Appendix

Multimedia Appendix 1 Psychosis and cannabis test quiz.

## Abbreviations

CBT: cognitive behavioral therapy
KT: knowledge translation
OR: odds ratio
PCT: psychosis and cannabis test
THC: delta-9-tetrahydrocannabinol
