# Supplementary material for: Online Knowledge Translation Program Involving Video Games and University Student–Led Tutorials About Cannabis and Psychosis for Black Youth: Mixed Method Feasibility Study
Source: JMIR Form Res. 2022 Jun 20;6(6):e33693. doi: 10.2196/33693 (PMC9253977; doi:10.2196/33693)
Supplement: Multimedia Appendix 1 [file formative_v6i6e33693_app1.docx]

**Multimedia Appendix 1.** Psychosis and cannabis test quiz.

**Question 1. What is psychosis?**

1. A condition that develops in people with criminal behaviours
2. A change in beliefs and emotions that distorts a person’s reality
3. An untreatable illness that destroys brain cells
4. A condition the person is born with that always develops in adolescence or adulthood
5. All of the above
   Answer B.

**Question 2. Which of the following is *true* of hallucinations and delusions?**

1. Hallucinations are false perceptions involving the senses (vision, smell, hearing or touch) and delusions are false beliefs that others do not share.
2. Hallucinations and delusions always occur together.
3. A delusion will eventually turn into a hallucination.
4. Delusions are false sensory perceptions and hallucinations are false beliefs.
5. None of the above.

Answer A.

**Question 3. What are possible symptoms of psychosis?**

1. Hallucinations and delusions
2. Loss of appetite, depressed mood, trouble sleeping
3. Feeling full of energy and blissfully happy
4. Having disturbing thoughts and emotions
5. All of the above

Answer E.

**Question 4. Among youth who are experiencing psychosis and also abusing substances; which of the following is *true*?**

1. Alcohol is the most commonly abused substance
2. Marijuana is the most commonly abused substance
3. Opioids are the most commonly abused substance
4. All of the above substances are abused similarly

Answer B.

**Question 5. Which of the following statements is *false*?**

1. Using a small amount of marijuana can create a sense of calm and euphoria
2. Using a large amount of marijuana can cause hallucinations and paranoia
3. Using a moderate amount of marijuana can cause difficulty with concentration and memory
4. Using a moderate amount of marijuana can improve performance in test-taking
5. All of the above false

Answer D.

**Question 6. Regular marijuana use during adolescence can increase the risk of developing psychosis by what proportion**:
(Regular use defined as three times a week or more for at least 3 months)

1. Four-fold compared to youth who do not use
2. It does not have any effect on the chance of developing psychosis
3. Doubles the risk compared to youth who do not use
4. It can actually decrease the chance of developing psychosis

Answer is C.

**Question 7. What are some signs of marijuana overuse Among Youth?**

1. Isolating from friends, personality changes, mood swings, anxiety
2. Increased attention to detail, more care given to physical appearance, decreased appetite
3. Improved judgement and driving skills
4. All of the above
5. None of the above

Answer B.

**Question 8. Which of the following chemicals found in strains of marijuana grown in North America, is associated with psychosis**

1. Cannabidiol (CBD)
2. Tetrahydrocannabinol (THC)
3. Researchers have not yet identified it.

Answer is B.

**Question 9. What is the best option, when a friend tells you that they’re hearing voices?**

1. Tell them to try to ignore the voices
2. Agree to keep their secret no matter what
3. Tell them to get angry at the voices, to intimidate them
4. Talk to them about seeing their family doctor

Answer D.

**Question 10: Which of the following services could provide help for someone experiencing psychosis associated with marijuana use?**

1. Early Intervention in Psychosis
2. Family Doctor
3. Crisis Outreach and Support Team
4. Substance Use Program for Youth
5. All of the above

Answer E.
